# Cell-Free microRNAs as Potential Oral Cancer Biomarkers: From Diagnosis to Therapy

**DOI:** 10.3390/cells8121653

**Published:** 2019-12-17

**Authors:** Óscar Rapado-González, Rafael López-López, José Luis López-Cedrún, Gabriel Triana-Martínez, Laura Muinelo-Romay, María Mercedes Suárez-Cunqueiro

**Affiliations:** 1 Department of Surgery and Medical-Surgical Specialties, Medicine and Dentistry School, Universidade de Santiago de Compostela, 15782 Santiago de Compostela, Spain; oscar.rapado@rai.usc.es; 2 Liquid Biopsy Analysis Unit, Translational Medical Oncology (Oncomet), Health Research Institute of Santiago (IDIS), 15706 Santiago de Compostela, Spain; 3 Instituto de Salud Carlos III, Centro de Investigación Biomédica en Red de Cáncer (CIBERONC), 28029 Madrid, Spain; 4 Translational Medical Oncology (Oncomet), Health Research Institute of Santiago (IDIS), Complexo Hospitalario Universitario de Santiago de Compostela (SERGAS), 15706 Santiago de Compostela, Spain; rafa.lopez.lopez@gmail.com; 5 Department of Oral and Maxillofacial Surgery, Complexo Hospitalario Universitario de A Coruña (SERGAS), 15006 A Coruña, Spain; lopezcedrun@centromaxilofacial.com; 6 Department of Radiation Oncology, Centro Oncológico de Galicia, 15009 A Coruña, Spain; gabriel.triana@cog.es; 7 Oral Sciences, Health Research Institute of Santiago de Compostela (IDIS), 15706 Santiago de Compostela, Spain

**Keywords:** microRNAs, oral cancer, liquid biopsy, biomarkers, epigenetic

## Abstract

Oral cavity cancer is the most frequent malignancy of the head and neck. Unfortunately, despite educational interventions for prevention and early diagnosis, oral cancer patients are often diagnosed in advanced stages associated with poor prognosis and life expectancy. Therefore, there is an urgent need to find noninvasive biomarkers to improve early detection of this tumor. Liquid biopsy has emerged as a valuable tool in medical oncology which provides new horizons for improving clinical decision making. Notably, cell-free microRNAs (miRNAs), a class of short non-coding RNAs, are emerging as novel noninvasive cancer biomarkers. Here, we provide an overview of the potential clinical application of cell-free miRNAs as diagnostic, prognostic, and therapeutic biomarkers in oral cancer.

## 1. Introduction

Currently, liquid biopsy has emerged as a novel tool in medical oncology that has provided a new horizon for improving clinical decision making. Tumors release several biomolecules into body fluids that could be used as biomarkers for diagnosis, prognosis, and therapy selection. Nowadays, analyses of tissue biopsies still remain the gold standard for diagnosis, however, these analyses hardly reflect tumoral heterogeneity [1]. Liquid biopsies provide information on the molecular landscape during tumor evolution through a noninvasive approach [2]. To this regard, the development of integrative clustering models combining molecular data from DNA, RNA such as microRNAs (miRNAs/miRs), and protein characterization [3] could provide greater accuracy of our understand of tumor heterogeneity, and therefore aid in the establishment of specific therapeutic strategies.

Oral cavity cancer is the most frequent malignancy of the head and neck, and the most common histological type is squamous cell carcinoma. Although the incidence and mortality rates observed for oral cancer vary according to geographic location and gender, a total of 354864 new cases and 177384 deaths have been estimated in 2018 worldwide [4,5]. Despite advances in cancer diagnosis and therapy, the five-year survival rate for oral cancer is still only approximately 50%, and the stage of disease at diagnosis is the most important prognostic factor for predicting survival [6]. While at early stages, the survival rate is approximately 89%, at late stages it decreases to 39% [7]. Unfortunately, oral cancer patients are still frequently diagnosed in advanced stages despite educational interventions for prevention and early diagnosis. Therefore, there is an urgent need for noninvasive biomarkers to improve the early detection.

Oral cancer is a multifactorial disease that results from a combination of genetic alterations and environmental risk factors, the most important of which are tobacco and alcohol consumption [8]. Human papillomavirus infection has also been associated with the etiology of oral cancer [9]. In addition, several genetic alterations in tumor suppressor genes (*APC*, *p53*), proto-oncogenes (Myc), oncogenes (Ras), and genes controlling normal cellular processes (EIF3E, GSTM1) have been involved in oral carcinogenesis [10]. Evidence shows that epigenetic alterations, such as DNA methylation, histone modifications, and non-coding RNA modifications (miRNAs) are major regulatory mechanisms in the development and progression of oral cancer [11].

MiRNAs are endogenous, single-stranded non-coding small RNAs of approximately 19 to 25 nucleotides, which regulate the gene expression at the post-transcriptional level by directly binding to the 3’ untranslated regions (UTR) of their target messenger RNA (mRNA) inducing mRNA degradation or inhibition of mRNA translation [12]. Through this mechanism, a single miRNA can target several mRNAs and a specific mRNA can be targeted by multiple miRNAs [13]. MiRNAs have a crucial role in the epigenetic regulation of cellular biological processes such as cell cycle regulation, differentiation, apoptosis, and migration. Their dysregulation is involved in the initiation and progression of human cancer [14,15]. Therefore, miRNAs can regulate the expression of target genes involved in cancer biology by acting as oncogenes or tumor suppressor genes [16]. Expression miRNA profiles of tumors and normal tissue are different. In fact, this expression can be specific to certain tumor types [17]. Several miRNA profiling studies have identified miRNA signatures associated with diagnosis, staging, prognosis, and response to therapy [18]. Notably, cell-free miRNAs are highly stable in body fluids because they are protected from endogenous RNase activity by encapsulation in lipoprotein complexes (apoptotic bodies, microvesicles, or exosomes), binding to ribonucleoprotein complexes (Argonaute-2 or nucleophosmin 1), or binding to high-density lipoproteins [19,20]. This biological advantage, together with their high sensitivity and specificity, have led to the acceptance of cell-free miRNAs as relevant cancer biomarkers.

In this review, we provide an overview of the potential clinical application of cell-free miRNAs as diagnostic, prognostic, and therapeutic biomarkers in oral cancer.

## 2. Origin of Cell-Free miRNAs in Oral Cancer

**Figure 1:**
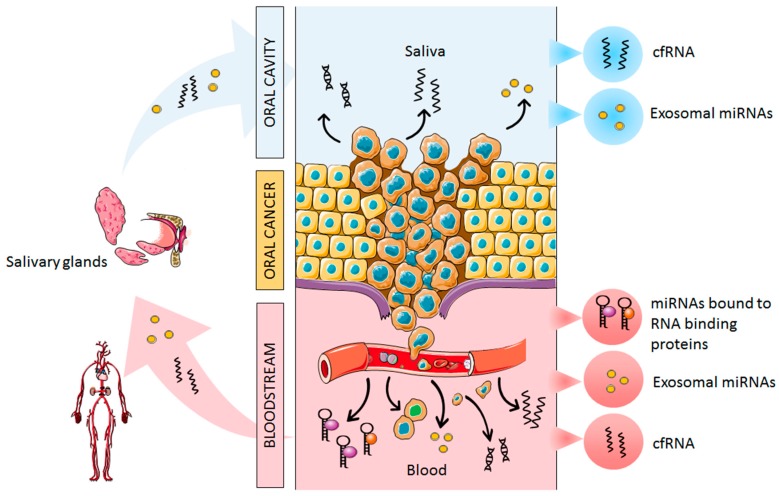
Schematic representation of cell-free miRNAs release into the bloodstream and saliva from oral cancer. Abbreviations: cfRNA, cell-free RNA.

During the carcinogenesis process, there is a direct release of tumor material into saliva and the bloodstream, detecting altered miRNA expression in several malignancies [21,22]. The release of miRNAs into the bloodstream and saliva is explained as a result of apoptotic and necrotic cell death and also by active cell secretion [19]. As we mentioned previously, circulating cell-free miRNAs are transported in the blood by different extracellular vesicles (apoptotic bodies and exosomes) [23], whereas the majority of circulating miRNAs are cofractionated with protein complexes such as Argonaute-2 [24]. In addition, oral tumors shed directly into saliva different markers, such as tumor DNA or miRNAs, which are secreted free or packed into extracellular vesicles, such as exosomes [25]. Moreover, tumor-derived miRNAs harbored in exosomes reach the salivary glands through the bloodstream and modify the saliva secretome as a result of tumor invasion (Figure 1).

## 3. MiRNAs as Diagnostic Biomarkers

Over the last 10 years, several researchers have investigated the clinical relevance of blood and saliva miRNAs as diagnostic markers in oral cancer.

### 3.1. Plasma and Serum

The first study that detected an altered expression of circulating miRNAs in oral cancer was performed by Wong et al. [26]. After a microarray expression analysis, overexpressed *miR-184* levels were validated in a cohort of 20 paired tongue squamous cell carcinoma and normal tissues. As in tissue, plasma levels of *miR-184* were significantly higher in cancer patients. Importantly, plasma *miR-184* levels were significantly decreased after tumor surgery which indicated a correlation between plasma and tissue *miR-184* levels. In addition, a functional assay revealed that *miR-184* enhanced the proliferation of tumor tongue cells and hindered the apoptotic process [26]. However, other authors [27,28] observed downregulated expression of *miR-184* in tissue from oral squamous cell carcinoma. Interestingly, *miR-184* expression levels were higher at surgical tumoral margin than at extra margin, indicating its potential for identifying minimal residual disease [27]. Other studies have described the potential oncogenic role of different miRNAs in oral cancer. Lin et al. [29] found significantly overexpressed *miR-24* plasma levels in oral cancer patients as compared with healthy controls, yielding an area under the receiver operating characteristic (ROC) curve (AUC) of 0.82 with 70% sensitivity and 82% specificity. Interestingly, upregulated circulating *miR-24* was also detected in tumoral oral tissue [29,30], showing a positive correlation with clinical stage [30]. Moreover, it has been demonstrated that *miR-24* promotes the proliferation, migration, and invasion of tongue squamous cell carcinoma by targeting FBXW7 [30]. Dysregulated circulating *miR-24* has been reported in other cancer locations, such as breast [31], colorectal [32], and lung [33] cancers. Other miRNAs have been described as oncogenic miRNAs in oral cancer, such as *miR-10b* [34] which was abnormally expressed in plasma of 54 oral cancer and seven patients with precancerous lesions as compared with 36 healthy controls. ROC analysis revealed a high discriminatory power for differentiating cancer (AUC = 0.932) and precancer (AUC = 0.967) patients with respect to healthy controls. In addition, oral squamous cell carcinoma xenograft mice models were developed, presenting significantly increased expression of *miR-10b* in plasma, which suggests its potential role in tumor formation. In vitro assays indicated that *miR-10b* was more highly associated with cell invasion and migration than tumor growth, although the regulatory pathway requires further research [35]. Importantly, circulating *miR-10b* has also been involved in other tumors, e.g., breast [36], pancreatic [37], and lung cancers [38]. Upregulated expression levels of circulating *miR-146a* were found in 51 oral cancer patients as compared with 12 healthy controls, differentiating cancer patients from controls with 79% sensitivity and 92% specificity (AUC = 0.86). Notably, after surgical tumor resection, circulating *miR-146a* levels were significantly decreased. In addition, results based on the xenograft model revealed that high plasma levels of *miR-146a* were associated with a higher tumor burden and the development of neck metastasis. Furthermore, this study showed a relationship between increased *miR-146a* expression and tumorigenesis and metastasis by the regulation of *IRAK1*, *TRAF6*, and *NUMB* gene expression [39].

Since early diagnosis is the key factor for improving the survival and quality of life of oral cancer patients, several studies have focused on identifying circulating miRNAs as potential biomarkers for early detection. Lu et al. [40] reported upregulated levels of *miR-196a* and *miR-196b* in plasma of patients with oral cancer and precancer with respect to healthy controls. ROC analysis indicated that circulating *miR-196a* and *miR-196b* could differentiate oral cancer patients from healthy individuals with AUC values of 0.864 and 0.960, respectively, and precancer patients from healthy individuals with AUC values of 0.760 and 0.840, respectively. Importantly, a logistic regression model was performed to validate the discriminatory power of both miRNAs between the two diseases groups and the control group with the following results: oral cancer vs. healthy controls presented 87.8% sensitivity and 92.5% specificity (AUC = 0.963), precancer vs. healthy controls showed 68.8% sensitivity and 84.9% specificity (AUC = 0.845), and disease group (oral cancer and precancer) vs. healthy controls presented 90.6% sensitivity and 84.9% specificity (AUC = 0.950). In addition, a positive correlation was found between *miR-196a* and *miR-196b* plasma expression levels, indicating that both miRNAs were upregulated in parallel during oral carcinogenesis. On the basis of these findings, circulating *miR-196a* and *miR-196b* seem to be important in early oral cancer stages, and therefore the combination of both miRNAs could be useful for the early detection of oral cancer. Previously, Liu et al. [41] evaluated the expression levels of *miR-196a* and *miR-196b* in tissue and plasma samples from oral cancer patients, finding significantly upregulated *miR-196a* levels. In addition, circulating *miR-196a* showed an 0.75 accuracy for distinguishing oral cancer patients from healthy controls. The human *miR-196* family (*miR-196a1*, *miR-96a2*, and *miR-196b*) plays a critical role in cancer development and pathogenesis by targeting several genes including *HOXB8*, *HMGA2*, and *Annexin A1* [42]. Upregulated circulating *miR-196a/b* expression levels have also been detected in gastric [43] and pancreatic cancer [44], indicating their involvement in various cancer types. Recently, Chang et al. [45] validated the expression levels of *miR-222-3p*, *miR-150-5p*, and *miR-423-5p* in 86 oral squamous cell carcinoma patients, 46 oral leukoplakia patients, and 50 healthy controls. Significant downregulation of *miR-222-3p* was found in oral leukoplakia patients, whereas *miR-423-5p* and *miR-150-5p* were significantly upregulated in oral cancer patients. For better differentiation, logistic regression models were performed using different miRNA combinations with the following results: *miR-150-5p* and *miR-222-3p* presented 90.91% sensitivity and 92.66% specificity for discriminating oral leukoplakia vs. healthy controls, *miR-150-5p* and *miR-423-5p* presented 70.91% sensitivity and 72.86% specificity for discriminating oral cancer vs. healthy controls, *miR-222-3p*, *miR-150-5p* and *miR-423-5p* presented 83.64% sensitivity and 85% specificity for discriminating oral leukoplakia vs. oral cancer, and 82.22% sensitivity and 90% specificity for discriminating oral leukoplakia vs. stage I oral cancer. Interestingly, functional enrichment analysis revealed the involvement of *miR-222-3p*, *miR-423-5p*, and *miR-150-5p* in different cancer-related pathways, such as Wnt, PI3K-Akt, MAPK, and Ras signaling pathway. These findings suggest the potential role of plasmatic miRNAs in oral carcinogenesis, as well as their high diagnostic value for early oral cancer detection. *MiR-221*/*miR-222* cluster has been shown to promote oral carcinogenesis by downregulation of PTEN [46]. As in oral cancer, upregulated circulating *miR-222* levels have been observed in a wide variety of tumors, e.g., pancreatic cancer [47] and glioma [48]. Unlike the above study by Chang et al. [45], Roy et al. [49] found that expression levels of *miR-423* were significantly downregulated in oral cancer tissue and can act as a tumor suppressor. Evidence shows a dual role of *miR-423* in cancer acting as an oncogene [50,51] or a tumor suppressor [52,53] in different malignancies. Moreover, dysregulated expression levels of *miR-150* have been reported in cancer, representing a potential diagnostic biomarker for acute myeloid leukemia [54], cholangiocarcinoma [55], and colorectal cancer [56].

Over the last few years, other circulating tumoral miRNAs have been highlighted as noninvasive biomarkers for oral cancer detection. In 2016, Tachibana et al. [57] identified 20 dysregulated miRNAs in plasma samples from gingival squamous cell carcinoma patients by profiling of 1211 human miRNAs using microarrays. Plasma *miR-223* was significantly upregulated in cancer samples as compared with controls, showing 67.7% sensitivity and 61.3% specificity. However, *miR-223* expression levels were downregulated in oral cancer tissues with respect to noncancerous tissues, suggesting that *miR-223* could be released into the bloodstream mainly from adjacent normal tissues as a biological defense mechanism to inhibit tumor growth. A functional analysis revealed that *miR-223* inhibited cell proliferation and inducted apoptosis in oral cancer cell lines, and also observed downregulated expression of *STMN1* and *IGF1R*. Xu et al. [58] reported significant upregulated serum *miR-483-5p* expression in 101 oral cancer patients with respect to 103 healthy controls. ROC analysis indicated that serum *miR-483-5p* could differentiate oral cancer from controls with 85% sensitivity and 74% specificity (AUC = 0.85). Interestingly, high serum *miR-483-5p* expression was positively correlated with lymph node metastasis and the Tumor-Node-Metastasis (TNM)-based staging system, allowing differentiation of late and early stages with 66% sensitivity and 78% specificity (AUC = 0.75). In addition, *miR-483-5p* was significantly upregulated in oral tumor tissues, suggesting that oral tumor cells could be shedding high levels of this miRNA into the blood during oral carcinogenesis. Similarly, Sun et al. [59] found significantly upregulated plasma levels of *miR-200b-3p* in oral squamous cell carcinomas with respect to healthy controls, observing significantly higher expression levels in grade II-III tumors than that of in grade I tumors. A significant decrease in circulating *miR-200b-3p* expression levels was observed after surgical removal of the tumor, reflecting its tumoral origin. The expression levels of *miR-200b-3p* were significantly upregulated in paired normal adjacent tissues as compared with tumoral tissue, suggesting that tumor cells can reduce the potential antitumorigenic behavior of this miRNA by releasing *miR-200b-3p* into the blood. The high sensitivity and specificity (90% and 88.75%, respectively) of *miR-200b-3p* indicates the diagnostic potential of this circulating biomarker for oral cancer detection. Plasma *miR-187-5p* has also been associated with oral carcinogenesis. Liu et al. [60] examined the expression of *miR-187-5p* in plasma samples of 63 oral squamous cell carcinoma patients and 23 healthy controls finding significantly upregulated levels in oral cancer and an AUC value of 0.73 to differentiate oral cancer patients from healthy controls. This high *miR-187-5p* expression was significantly decreased after tumor resection, indicating that circulating *miR-187-5p* came from the tumor. Importantly, *miR-187-5p* could act as a potential oncogene promoting proliferation, migration, and anchorage-independent colony formation of oral cancer cells. Recently, Lu et al. [61] selected a set of five miRNAs (*miR-99a-5p*, *miR-138-5p*, *miR-375-3p*, *miR-21-5p*, and *miR-31-5p*) based on previous miRNA profiling studies and analyzed their expression levels in tissue and serum samples. Although only *miR-31-5p* was significantly upregulated in tissue and serum samples, the serum expression levels of *miR-99a-5p*, *miR-138-5p* and *miR-375-3p* were significantly related to clinical stage. On the basis of ROC analysis, serum *miR-31-5p* could discriminate oral cancer from healthy controls with an AUC value of 0.661, whereas the model including the five serum miRNAs showed an AUC of 0.776, with 76.8% sensitivity and 73.6% specificity. These findings suggest the diagnostic potential of circulating *miR-31-5p* as an independent biomarker for oral cancer detection. Altered expression of circulating *miR-31* has been reported in oral cancer patients previously by Liu et al. [62] In their study, *miR-31* expression was significantly upregulated in plasma from cancer patients as compared with healthy controls, showing an AUC value of 0.82. Plasma *miR-31* expression levels were significantly decreased after surgery, suggesting that *miR-31* is released into the bloodstream from the tumor [62]. Circulating *miR-21* which is dysregulated in many cancer types was also significantly overexpressed in oral cancer [63,64,65].

Because the origin of extracellular miRNAs remains unclear, other large-scale miRNA profiling studies have been performed to identify novel miRNA expression patterns in tumoral tissue and the bloodstream. Rabinowits et al. [66] compared the miRNAs expression profile of benign and malignant tongue tissue and plasma from five tongue squamous cell carcinoma patients by microarrays. Out of the 359 miRNAs detected, 16 miRNAs (nine upregulated and seven downregulated) were differentially expressed between cancer and noncancer tissue. As in the tumor, *miR-19a*, *miR-27b*, *miR-20a*, *miR-28-3p*, *miR-200c*, *miR-151-3p*, *miR-223*, and *miR-20b* were upregulated in plasma, free and encapsulated in exosomes. On the other hand, *miR-512-3p* was only associated with exosomes. However, of the seven tumor downregulated miRNAs, four miRNAs (*miR-370*, *miR-139-5p*, *miR-let-7e*, and *miR30c*) were expressed in plasma, free and within exosomes, and one miRNA (*miR-516-3p*) was not detected, and two miRNAs (*miR-22* and *miR-145-3p*) were associated with exosomes only. These results indicate that tumor miRNAs are present in the bloodstream as cell-free miRNAs and exosomal-miRNAs, however, exosomal-miRNAs could better reflect the tumor miRNA profile. Schneider et al. [67] compared the miRNA profile in tissue and serum samples of five oral squamous cell carcinoma patients by RNA sequencing. After bioinformatic analysis, a total of 255 miRNAs were identified in tissue (cancer and noncancer) and 381 miRNAs were detected in serum; 214 miRNAs were present in both tissue and plasma. Importantly, out of the 48 miRNAs found to be significantly dysregulated in cancer tissue as compared with normal tissue, 30 were also detected in serum; indicating that most tumor miRNAs can be detected in circulation and are potential diagnostic biomarkers for cancer. Recently, Pedersen et al. [68] reported a thorough description of the miRNome in both oral squamous cell carcinoma and normal oral mucosa. Using miRNA-seq data of oral cancer tissues, expression levels of a set of miRNAs were analyzed in plasma samples from 55 oral cancer patients and 18 healthy controls. Only four miRNAs (*miR-30a-5p*, *miR-370-3p*, *miR-144-5p*, and *miR-769-5p*) were significantly upregulated in plasma from cancer patients as compared with healthy controls. Interestingly, a model generated by the combination of plasma *miR-30a-5p* and *miR-370-3p* yielded an AUC value of one, indicating its high discriminatory power for oral cancer detection. However, these predictive miRNAs were different between plasma and tissue. The authors considered that the discordance between tissue and plasma miRNAs could be the result of the systemic response to oral cancer. 

### 3.2. Saliva

Nowadays, saliva represents a revolutionary liquid biopsy in many fields of science, including systemic diseases, oral diseases, and pharmacotherapy. Saliva is a complex body fluid that contains diverse types of biomolecules, including a wide variety of enzymes, hormones, antibodies, antimicrobial constituents, and growth factors [69]. Saliva is considered an ultrafiltrate of the blood, so most of the molecules present in the blood are detected in saliva. These molecules enter into saliva by several mechanisms such as transcellular routes (passive or active transport), paracellular routes (extracellular ultrafiltration) or through the gingival sulcus [70]. Therefore, saliva analysis represents an opportunity to detect biomarkers associated with the onset and development of local [71] and distant [72] diseases. Therefore, saliva is considered “the mirror of the body” [73]. In addition, saliva presents numerous advantages as a diagnostic medium because its collection is simple, noninvasive, and cost-effective. This makes it an attractive biofluid for screening, diagnosis, and monitoring of disease.

Since saliva originates in the oral cavity and oral tissues are immersed it, this biofluid constitutes a direct source of biomarkers for oral cancer detection. Park et al. [74] carried out one of the first studies that aimed to determine miRNA profile in whole and supernatant saliva samples from healthy controls and profiled a total of 314 miRNAs. Although whole and supernatant saliva showed similar profiles, a major heterogeneity was present in the whole saliva which could be due to the presence of miRNAs derived from desquamated oral squamous cells. In addition, the same authors compared the expression profile of four miRNAs (*miR-142-3p*, *miR-93*, *miR-125a*, and *miR-200a*) in a cohort of 50 oral squamous cell carcinoma patients and 50 healthy controls, observing significantly decreased expression of *miR-125a* and *miR-200a* in supernatant saliva of cancer patients. The downregulation of *miR-200* family, a group of tumor suppressor miRNAs, promoted epithelial-mesenchymal transition of oral tumor cells through the upregulation of *ZEB1* and *ZEB2* genes and increased vimentin expression [75]. Other miRNAs have been identified as regulators in the development and progression of oral cancer [76,77,78]. Oncogenic behavior is presented by *miR-31* in several malignancies, promoting proliferation and invasion of oral cancer cells. Liu et al. [79] explored the potential of *miR-31* as a clinical biomarker for oral squamous cell carcinoma, and they found significantly upregulated *miR-31* levels in cancer patients as compared with healthy controls, both in saliva and plasma. Moreover, a significant decrease in salivary and plasma *miR-31* levels was observed after tumor resection, which suggests that *miR-31* was released from the tumor into the bloodstream and later into saliva. Importantly, a significant positive correlation between saliva and plasma *miR-31* levels was observed. However, *miR-31* expression levels were significantly higher in saliva with respect to plasma, indicating major local secretion of *miR-31* from oral squamous cell carcinoma cells, perhaps as a consequence of the direct contact between saliva and the oral tumor. These results revealed the potential application of salivary *miR-31* as a sensitive biomarker for detecting oral cancer. Epithelial *miR-31* upregulation and epithelial dysplasia were described as independent factors for oral potentially malignant disorders, which presented increased *miR-31* expression levels. Significant *miR-31* upregulation was also detected in saliva samples of oral potentially malignant disorder patients as compared with healthy controls (AUC = 0.76), although no association was found with epithelial dysplasia. These data evidenced the potential role of *miR-31* in the early steps of oral cancer development [80]. Other miRNAs, such as *miR-21* and *miR-184*, showed significantly increased levels in saliva from oral squamous cell carcinoma and oral potentially malignant disorders, while salivary *miR-145* showed a significant decrease. However, only *miR-184* made it possible to discriminate between oral squamous cell carcinoma and oral potentially malignant disorders with dysplasia [81].

Advances in high-throughput screening techniques have allowed researchers to characterize salivary miRNoma. Momen-Heravi et al. [82] analyzed a total of 734 human miRNAs by using NanoString nCounter and identified 13 significantly dysregulated miRNAs in saliva samples of oral squamous cell carcinoma as compared with healthy controls, including 11 downregulated miRNAs (*miR-136*, *miR-147*, *miR-1250*, *miR-148a*, *miR-632*, *miR-646*, *miR-668*, *miR-877*, *miR-503*, *miR-220a*, and *miR-323-5p*) and two upregulated miRNAs (*miR-24* and *miR-27b*). In addition, *miR-27b* and *miR-136* gave an AUC of 0.964 and 0.963, respectively, for differentiating cancer patients from healthy controls. Of note, *miR-27b* could play a tumor-suppressor role in oral cancer by targeting Frizzled7 and Wnt signaling pathway, which are involved in cell proliferation, differentiation, migration, and invasion of oral cancer cells [83]. Another study identified 419 significantly dysregulated miRNAs in tongue squamous cell carcinoma saliva samples as compared with healthy controls using microarrays. Furthermore, expression levels of two upregulated miRNAs (*miR-33a-3p* and *miR-198*), and downregulated *miR-139-5p* were selected for validating in a cohort of 25 cancer patients and 25 healthy controls, observing significant downregulation of *miR-139-5p* in tumor samples. Moreover, after tumor surgery, this study evaluated the expression levels of *miR-139-5p*, which reached levels comparable to those of healthy controls. According to a ROC curve analysis, *miR-139-5p* made it possible to differentiate cancer patients from healthy individuals with an AUC of 0.805 [84]. Similarly, Chen et al. [85] found upregulated expression of *miR-139-5p* in normal tissue as compared with tissue from tongue squamous cell carcinoma, which could suggest the origin of salivary miR-139-5p.

Like plasma and serum, salivary extracellular vesicles currently represent a strong source of biomolecules from oral tumors because of protection from enzyme degradation [86]. Therefore, the isolation of salivary extracellular vesicles constitutes an attractive approach for identifying tumor-derived miRNAs. Thus, Gai et al. [87] provided the first characterization of the miRNA profile in salivary extracellular vesicles from oral cancer patients. *MiR-320-3p* and *miR-517b-3p* were expressed only in salivary extracellular vesicles from oral cancer patients while *miR-412-3p* and *miR-512-3* were significantly increased in cancer patients as compared with healthy controls, presenting AUC values of 0.847 and 0.871, respectively. Importantly, functional analysis revealed the involvement of these miRNAs in different pathways normally activated in oral cancer, such as TGFβ and ErbB signaling pathways. These findings indicated that salivary extracellular vesicles could harbor specific molecular biomarkers with potential for diagnosing oral cancer. Recently, another study focusing on extracellular vesicles isolated from oral swirls developed an algorithm-based risk classification of five miRNAs (*miR-24-3p*, *miR-21-5p*, *miR-99a-5p*, *let-7c-5p*, and *miR-100-5p*) discriminating normal mucosa and oral cancer with an AUC of 0.867. In addition, this dysregulation score showed significant statistical differences between non-oral cancer (histological normal epithelia and oral potentially malignant disorders) from oral cancer. Therefore, this miRNA signature constitutes a promising noninvasive screening tool [88] (Table 1).

It is important to highlight that some previously mentioned salivary miRNAs have also been described as dysregulated in other malignancies and inflammatory diseases. In this sense, salivary *miR-21* was significantly upregulated in remote tumors (colorectal [89], oesophageal [90,91], and pancreatic [92]) and chronic inflammation (ulcerative colitis [93] and oral lichen planus [94]). Other salivary miRNAs, such as *miR-31*, *miR-125a*, and *miR-27b* were significantly altered in oral potentially malignant disorders [94,95]. These findings indicate that some miRNAs represent biomarkers for potentially malignant lesions and cancer; however, individually they do not necessarily identify the anatomic origin of the systemic disease nor do they discriminate potential malignancy from cancer.

## 4. MiRNAs as Prognostic Biomarkers

Another important clinical utility of circulating miRNAs in cancer is their ability to provide information about clinical patient outcome during disease course or after therapeutic intervention. This could make it possible to identify those patients at highest risk for cancer recurrence. Several tissue and circulating miRNAs have been put forward as prognostic predictors in oral cancer [58,96]. To the best of our knowledge, the first study that evaluated prognostic markers in liquid biopsy was by Liu et al. [41], observing increased plasma *miR-196a* levels associated with poor disease-free survival. Xu et al. [58] examined the expression levels of *miR-483-5p* in serum samples from 85 oral cancer patients who underwent chemotherapy after surgery. High serum *miR-483-5p* expression was significantly associated with shorter overall survival, poorly differentiated tumors, late-stage and lymph node metastasis. Serological *miR-9* was downregulated in oral cancer as compared with oral leukoplakia patients and healthy controls, showing a significant association with different clinicopathological variables, including the T stage, lymph node metastasis, and TNM stage. Furthermore, low circulating *miR-9* levels were significantly associated with poor disease-free and overall survival [97]. In another study, a multivariate logistic regression analysis showed that *miR-200b-3p* was an independent prognostic predictor, as were old age and cancer grade [59]. Interestingly, Li et al. [60] analyzed the expression levels of *miR-187-5p* in oral cancer patients before and after tumor resection, observing that postoperative patients with decreased *miR-187-5p* levels presented a better prognosis than those with increased expression. These results indicated that circulating *miR-187-5p* represents a potential biomarker for predicting tumor recurrence. In the same line, downregulated plasma levels of *miR-486-5p*, *miR-375* and *miR-92b-3p* were significantly associated with oral cancer recurrence after surgery, indicating their potential for monitoring the risk of recurrence [98]. More recently, Chen et al. [99] analyzed the expression level of *miR-99a* in serum samples from oral cancer patients and observed significantly lower levels as compared with healthy individuals. Interestingly, low serum *miR-99a* levels were significantly associated with shorter overall and disease-free survival, as well as being negatively correlated with TNM stage and histological grade. According to univariate and multivariate analyses, TNM stage, histological grade, and serum *miR-99a* expression were all significant independent prognostic factors for predicting survival. In contrast to these studies that only evaluated the prognostic value of a single circulating miRNA, Shi et al. [100] were the first to report a miRNA-signature for predicting survival in oral cancer. This two-miRNA signature (*miR-626* and *miR-5100*) was independently associated with shorter overall and disease-free survival. Moreover, a prognostic model integrating TNM stage and miRNA signature improved prognostic ability as compared with TNM stage or miRNA signature alone.

Although some studies have reported the prognostic value of circulating miRNAs, it is also essential to establish markers with predictive power for patient management. Predictive markers provide information about sensitivity and resistance to a specific type of therapy, making it possible to identify those patients who most likely will benefit from therapy. In this sense, knowing beforehand if a patient will have a favorable or unfavorable response to therapy could help clinicians determine the best drug or combination of drugs for that individual, thus, avoiding ineffective therapy and drug-related toxicities. To date, only one study has described the potential predictive value of circulating miRNAs for evaluating therapeutic efficacy in oral cancer. In this study, Nakashima et al. [101] evaluated the prognostic value of *miR-1290* in oral cancer patients who underwent preoperative 5-fluorouracil–based chemoradiotherapy, observing that patients with low circulating *miR-1290* expression levels had shorter overall and disease-free survival as compared with patients with high circulating *miR-1290* expression levels. Interestingly, patients with low miR-1290 levels showed lower pathological differentiation and poor response for preoperative chemoradiotherapy, which indicates that circulating *miR-1290* is related to tumor characteristics and resistance to chemoradiotherapy. These findings provide evidence regarding the potential of circulating therapy-responsive miRNAs as noninvasive predictive and prognostic biomarkers for oral cancer patients undergoing chemoradiotherapy (Table 2).

## 5. MiRNAs as Therapeutic Targets

Because miRNAs can target multiple mRNAs involved in cancer development and progression, therapeutic approaches based on miRNAs represent a promising novel strategy for cancer therapy. Therapeutic miRNA approaches are based on miRNA mimics (miRNA-replacement therapy) and anti-miRs (miRNA-silencing therapy). The aim of miRNA-mimic approaches is to replace tumor suppressor miRNAs using synthetic double-stranded small RNA molecules with identical sequence and function to mature endogenous miRNAs. Interestingly, it could also be possible to restore the expression of a tumor suppressor miRNA by inserting genes coding for miRNAs into viral constructs, such as adenovirus-associated vectors. The aim of anti-miRs approaches is to silence oncogenic miRNAs using anti-miR oligonucleotides, miRNA sponges, miRNA masking, and small RNA inhibitors [102,103]. Anti-miR oligonucleotides are chemically modified antisense oligonucleotides. These include single-stranded RNA analogues 2′-*O*-methyl group-modified nucleotides, 2′-*O*-methoxyethyl group-modified nucleotides (also called antago-miRs), and locked nucleic acids anti-miR constructs, which have been designed to bind to mature miRNA guide strands in order to block target miRNA expression [104]. Another strategy that has been described to inhibit miRNA expression is miRNA sponge. This method is based on transcripts with multiple complementary binding sites to specific miRNAs, making it possible to block even an entire family of related miRNAs [105]. In addition, blocking oncogenic miRNAs can be achieved by miRNA-mask oligonucleotides that consist of synthetic single-stranded 2′-*O*-methyl-modified antisense oligonucleotides complementary to the 3’ UTR of the target mRNA for de-repressing its target gene [106]. For a similar purpose, the “SMIR-approach” has emerged. This novel inhibitory-miRNA therapy is based on small molecule inhibitors of miRNAs that are known to be able to interact with RNA, inhibiting miRNA biogenesis or impeding miRNA-target interaction [107].

Most of the reported studies in the present review have shown promising results of miRNA-based on therapeutic approaches in vitro or in vivo. Thus, Wong et al. [26] demonstrated that *miR-184* inhibitor reduced the proliferation rate in three different tongue squamous cell carcinoma cell lines. Lin et al. [29] observed that the blockage of *miR-24* expression by anti-sense *miR-24* locked nucleic acid yielded a decrease of endogenous *miR-24* expression levels, inhibiting the growth of SAS oral cancer cell lines. Similarly, *miR-146a* locked nucleic acid decreased the proliferation rate of oral cancer cells in vitro. Interestingly, when *miR-146a* locked nucleic acid and scramble control locked nucleic acid were complexed with atelocollagen and injected into xenografic tumor mouse model, a significant decrease in tumoral growth was observed, which suggests that *miR-146a* locked nucleic acid represses the oncogenicity of oral cancer cells [39]. Another potential candidate for miRNA-therapy in oral cancer is *miR-187*. Liu et al. [60] described circulating *miR-187-5p* as a potential oncogene in oral cancer and they found that miR-187-5p mimic increased exogenous miR-187-5p levels but did not alter *miR-187-3p* expression, demonstrating the high specificity of *miR-187-5p* mimic. Exogenous *miR-187-5p* expression was associated with an increase in the growth of SAS oral cancer cells, the migration of SAS and OECM1 oral cancer cells, and anchorage-independent colony formation, suggesting that the blockage of *miR-187-5p* has therapeutic efficacy against the progression of oral cancer. Recent research has demonstrated that *antagomiR-31-5p* significantly inhibited the proliferation of UM1 oral cancer cells and *miR-31-5p* mimics significantly enhanced the proliferation of normal epithelial HaCaT cells, indicating the oncogenic role of *miR-31-5p* in oral carcinogenesis. The inhibition of oral tumor progression has also been confirmed in models involving mice undergoing *antagomiR-31-5p* therapy with xenograft derived from oral cancer patients. These authors observed that *antagomiR-31-5p* significantly delayed the tumor growth of oral cancer patient-derived xenografts. In addition, they found significantly reduced p-AKT levels and increased PTEN levels in *antagomiR-31-5p* treated xenografts, indicating that *miR-31-5p* could be a therapeutic target in oral cancer via the PTEN/AKT pathway [61].

## 6. Conclusions: Future Perspectives and Challenges

Over the last few years, ongoing research has highlighted the significant clinical potential of cell-free miRNAs in a variety of human diseases, including cancer. Scientific evidence supports the interest of cell-free miRNAs for diagnosis and prognosis in oral cancer. Nevertheless, it is still necessary to elucidate the functional pathways and genetic networks associated with each dysregulated miRNA in order to understand their role in oral cancer development and progression.

As we have reported previously, several dysregulated cell-free miRNAs have been found in saliva and blood from oral cancer patients. However, a number of challenges still remain to be addressed before the clinical implementation of miRNAs becomes feasible. First, the scientific community should establish standardized protocols for body fluid collection and miRNA isolation, quantification, and analysis. Secondly, there is an urgent need to validate cell-free miRNA signatures with the highest sensitivity and specificity for oral cancer in particular. In this sense, multicenter studies based on large case-control designs of patients should be carried out to allow cross-comparisons. Thirdly, it is necessary to determine the most suitable biofluid (saliva vs. blood) for analyzing the miRNA profile in oral cancer. Comprehensive studies would make it possible to identify the most commonly altered miRNAs present in saliva and blood and to discover specific molecular patterns in both biofluids. Finally, although miRNAs have shown potential as therapeutic targets in oral cancer, research in this field is still in its infancy, thus, further research into specific miRNA therapy approaches should be carried out. In addition, the benefit of cell-free miRNAs for treatment monitoring and for detecting minimal residual disease in oral cancer should be elucidated. Advances in understanding the role of cell-free miRNAs in cancer will help researchers to achieve this goal.

In the near future, advances in high-throughput technology would help to better explore omics. Molecular signatures based on a combination of several cell-free miRNAs with analytes from other omics would also contribute to developing multi-omic panels for improving precision oncology. In the long-term, the use of big data would also permit large accurate molecular profiles for each patient yielding a new cancer stratification concept that we have coined “molecular cancer staging”. To attain this goal, we need a new health system model that brings together professional skills from a wide variety of scientific fields including bioinformatics, biology, mathematics, and biotechnology. This represents a new paradigm for precision oncomedicine.

In summary, cell-free miRNA profiles represent a promising noninvasive approach for oral cancer diagnosis, prognosis, and therapeutic targets. Evidence exists supporting the potential clinical utility of cell-free miRNAs in oral cancer, and future research efforts should focus on finding the most accurate miRNA panel for improving patient management.

## Figures and Tables

**Table 1:** Salivary cell-free miRNAs as diagnostic biomarkers in oral cancer.

Study	Preanalytical Variables	RNA Extraction	Study Cohort	Technique	Molecular Profile (oral cancer vs. HC)	Sensitivity/Specificity (%)	AUC
Park et al., 2009 [74]	Cell-free saliva Preservation: SUPERase·In (Ambion)	Volume: 400 μL Kit: mirVana miRNA Isolation (Ambion)	50 OSCC 50 HC	RT-preamp-qPCR	*miR-200a* (↓) *miR-125a* (↓) *miR-200a* + *miR-125a* (↓)	ND	0.65 0.62 0.66
Liu et al., 2012 [79]	Cell-free saliva Storage: –80 °C	Volume: 600 µL Kit: mirVana PARIS Isolation (Ambion)	45 OSCC 10 OVL 24 HC	TaqMan qRT-PCR	*miR-31* (↑)	80/68	0.82
Momen-Heravi et al., 2014 [83]	Cell-free saliva Centrifugation: 2600 × *g* 15 min at 4 °C Preservation: 5 µL of SUPERase·In (Ambion) per milliliter of supernatant Storage: −80 °C	Volume: 440 µL Kit: RNeasy (Qiagen)	9 OSCC 8 OSCC-R 8 LP 9 HC	TaqMan qRT-PCR	*miR-27b* (↑) *miR-136* (↓)	85.71/100 88.89/100	0.964 0.968
Zahran et al., 2015 [81]	Cell-free saliva Centrifugation: (i) 2500 × g 10 min at 4 °C, (ii) 10,000 × g 1 min	Volume: 200 µL Kit: miRNeasy serum/plasma extraction (Qiagen)	20 OSCC 40 OPMD 20 RAS 20 HC	SYBR Green qRT-PCR	*miR-21* (↑) *miR-145* (↓) *miR-184* (↑)	NS	NS
Duz et al., 2016 [84]	Cell-free saliva Centrifugation: 2600 × g 15 min at 4 °C Storage: −80 ◦C	Volume: NS Kit: mirVana PARIS (Ambion)	25 TSCC 25 HC	TaqMan qRT-PCR	*miR-139-5p* (↓)	73.9/85	0.805
Gai et al., 2018 [87]	Cell-free saliva Centrifugation: 2600 × g 15 min at 4 °C Storage: −80 ◦C	Volume: 250 µL Kit: mirVana Isolation (Thermo Fisher Scientific)	21 OSCC 11 HC	SYBR Green qRT-PCR	*miR-512-3p* (↑) *miR-412-3p* (↑)	ND	0.847 0.871
Yap et al. 2019 [88]	Cell-free oral swirls Centrifugation: 4000 × g 4 min at 4 °C Storage: −20 °C	Volume: NS Kit: mirVana Isolation (Life Technologies)	53 OSCC 54 NMA 9 HNE 74 OPMD	qRT-PCR	*miR-24-3p* + *miR-21-5p* + *miR-99a-5p* + let-7c-5p + *miR-100-5p* (↑)	NS	0.867

Abbreviations: OSCC, oral squamous cell carcinoma; HC, healthy controls; OVL, oral verrucous leukoplakia; OSCC-R, oral squamous cell carcinoma in remission; OLP, oral lichen planus; OPMD, oral potentially malignant disorders; RAS, recurrent aphthous stomatitis; TSCC, tongue squamous cell carcinoma; HNE, histologically normal epithelium; NMA, no mucosal abnormalities; qRT-PCR, quantitative real-time polymerase chain reaction; NS, not specified-unclear; ND, no data; (↑), upregulated; (↓) downregulated.

**Table 2:** Circulating miRNAs as biomarkers in oral cancer.

Study	Preanalytical Variables	RNA Extraction	Study Cohort	Technique	Molecular Profile (oral cancer vs. HC)	Sensitivity/Specificity (%)	AUC	Clinical Application
Wong et al., 2008 [26]	Plasma	Volume: NS Kit: mirVana miRNA Isolation (Ambion)	30 TSCC 38 HC	TaqMan qRT-PCR	*miR-184* (↑)	ND	ND	Diagnosis
Lin et al., 2010 [29]	Plasma	Volume: NS Kit: NS	33 OSCC 10 HC	qRT-PCR	*miR-24* (↑)	70/92	0.82	Diagnosis
Liu et al., 2010 [62]	Plasma	Volume: 600 µL Kit: mirVana PARIS Isolation (Ambion)	43 OSCC 21 HC	TaqMan qRT-PCR	*miR-31* (↑)	ND	0.82	Diagnosis
Lu et al., 2012 [35]	Plasma	Volume: 200 µL Kit: miRNeasy Mini (Qiagen)	54 OSCC 7 OLK 36 HC	TaqMan qRT-PCR	*miR-10b* (↑)	94.4/80	0.932	Diagnosis
Hung et al., 2013 [39]	Plasma	Volume: NS Kit: NS	51 OSCC 12 HC	TaqMan qRT-PCR	*miR-146a* (↑)	79/92	0.86	Diagnosis
Liu et al., 2013 [41]	Plasma	Volume: NS Kit: mirVana PARIS (Ambion)	65 OSCC 24 HC	TaqMan qRT-PCR	*miR-196a* (↑)	ND	0.75	Diagnosis and prognosis
Lu et al., 2015 [40]	Plasma	Volume: 200 µL Kit: miRNeasy mini (Qiagen)	90 OC 16 OPMD 53 HC	TaqMan qRT-PCR	*miR-196a* (↑) *miR-196b* (↑) *miR-196a* + *miR-196b* (↑)	66.7/96.2 97.8/81.1 87.8/92.5	0.864 0.960 0.963	Diagnosis
Xu et al., 2016 [58]	Serum Storage: −80 °C	Volume: NS Kit: miRNeasy RNA Isolation (Qiagen)	101 OSCC 103 HC	SYBR Green qRT-PCR	*miR-483-5p* (↑)	85.3/74.6	0.85	Diagnosis and prognosis
Sun et al., 2016 [97]	Serum Centrifugation: 3500 × g 5 min Storage: −80 °C	Volume: NS Kit: miRNeasy (Qiagen)	104 OSCC 30 OLK 40 HC	SYBR Green qRT-PCR	*miR-9* (↓)	ND	ND	Diagnosis and prognosis
Tachibana et al., 2016 [57]	Plasma Storage: −80 °C	Volume: 100 μL Kit: miRNeasy (Qiagen)	31 GSCC 31 HC	qRT-PCR	*miR-223* (↑)	67.7/61.3	0.703	Diagnosis
Liu et al., 2017 [60]	Plasma	Volume: NS Kit: mirVana PARIS Isolation (Ambion)	63 OSCC 26 HC	qRT-PCR	*miR-187-5p* (↑)	ND	0.73	Diagnosis and prognosis
Chang et al., 2018 [45]	Plasma Centrifugation: 3000 × g 10 min Storage: −80 °C	Volume: 200 µL Kit: miRNeasy Serum/Plasma (Qiagen)	70 HC 66 OLK 114 OSCC	SYBR Green qRT-PCR	*miR-150-5p* (↑) *miR-423-5p* (↑) *miR-150-5p* + *miR-423-5p* (↑)	60.55/77.14 58.72/72.86 70.91/72.86	0.702 0.677 0.749	Diagnosis
Sun et al., 2018 [59]	Plasma Storage: −80 °C	Volume: 200 μL Kit: miRNeasy Mini (Qiagen)	80 OSCC 80 HC	TaqMan qRT-PCR	*miR-200b-3p* (↑)	90/88.75	0.917	Diagnosis and prognosis
Chen et al., 2018 [99]	Serum Centrifugation: 3500 × g 5 min Storage: −80 °C	Volume: NS Kit: TRIzol Reagent	121 OSCC 55 HC	TaqMan qRT-PCR	*miR-99a* (↓)	80.2/83.6	0.911	Diagnosis and prognosis
Pedersen et al., 2018 [68]	Plasma	Volume: NS Kit: miRCURY RNA isolation (Exiqon)	55 OSCC 15 HC	TaqMan qRT-PCR	*miR-30a-5p* (↑) *miR-370-3p* (↑) *miR-144-5p* (↑) *miR-769-5p* (↑) *miR-30a-5p* + *miR-769-5p* (↑)	ND	0.97 ND ND 0.94 1	Diagnosis
Mahmood et al., 2019 [65]	Plasma	Volume: NS Kit: Favorgen Nucleic acid extraction	100 OSCC 100 HC	SYBR Green qRT-PCR	*miR-21* (↑)	91/54	0.829	Diagnosis
Nakashima et al., 2019 [101]	Plasma	Volume: NS Kit: miRNeasy Serum/Plasma (Qiagen)	55 OSCC 10 HC	SYBR Green qRT-PCR	*miR-1290* (↓)	ND	ND	Diagnosis, prognosis and predictive resistance to therapy
Lu et al., 2019 [61]	Serum Centrifugation: (i) 4000 rpm 10 min 4 °C, (ii) 12,000 rpm 15 min 4 °CStorage: −80 °C	Volume: NS Kit: miRcute miRNA Isolation (Tiangen Biotech)	82 OSCC 53 HC	SYBR Green qRT-PCR	*miR-31-5p* (↑)	69.8/52.4	0.661	Diagnosis and therapeutic target
Shi et al., 2019 [100]	Serum	Volume: 200 µL Kit: miRNeasy Mini Kit (Qiagen)	218 OSCC 90 HC	TaqMan qRT-PCR	*miR-626* (↑) *miR-5100* (↑)	76.8/77.3	0.771	Prognosis

Abbreviations: OSCC, oral squamous cell carcinoma; HC, healthy controls; TSCC, tongue squamous cell carcinoma; GSCC, gingival squamous cell carcinoma; OLK, oral leukoplakia; OC, oral cancer; OPMD, oral potentially malignant disorders; qRT-PCR, quantitative real-time polymerase chain reaction; (↑), upregulated; (↓), downregulated.
